# Myopathies Associated With Monoclonal Gammopathies of Clinical Significance: A Narrative Review

**DOI:** 10.7759/cureus.100388

**Published:** 2025-12-30

**Authors:** Mohamed Reda Belkhribchia

**Affiliations:** 1 Department of Neurology, Hassan II Regional Hospital, Dakhla, MAR

**Keywords:** acquired glycogen storage myopathy, al amyloidosis, autologous stem cell transplantation, chemotherapy, monoclonal gammopathies of clinical significance, myopathy, sporadic late-onset nemaline myopathy

## Abstract

Monoclonal gammopathies of clinical significance (MGCS)-associated myopathies are rare diseases in which the monoclonal protein (M-protein) causes significant muscle damage. The pathophysiology of MGCS-associated myopathies remains unclear. A toxic M-protein may cause muscle degradation directly through deposition of its light and/or heavy chains in the muscle or indirectly via complex dysregulation of the immune system. Among the MGCS-associated myopathies, amyloid light chain (AL) amyloidosis-associated myopathy and sporadic late-onset nemaline myopathy with monoclonal gammopathy (SLONM-MG) are recognized. However, non-amyloid light chain deposition disease (LCDD)-associated myopathy and the recently described MGCS-associated glycogen storage myopathy (GSM) are also rare entities belonging to the array of MGCS-associated myopathies. In AL amyloidosis-associated myopathy and non-amyloid LCDD-associated myopathy, the muscle involvement usually occurs in the context of a broader systemic disease. Whereas, in SLONM-MG and MGCS-GSM, no systemic involvement occurs outside the muscle (skeletal and cardiac muscle). The confirmation of these myopathies relies on distinctive pathological features in muscle biopsy studies. Since these acquired myopathies are linked to MGCS, many authors consider that chemotherapy is the best therapeutic approach to manage these myopathies. Despite their severity, an early and accurate diagnosis is crucial due to the treatability of MGCS-associated myopathies.

## Introduction and background

Monoclonal protein (M-protein) is a monoclonal immunoglobulin produced by an abnormal clone of plasma cells in bone marrow, which serves as a key diagnostic marker for various plasma cell disorders ranging from benign monoclonal gammopathy of undetermined significance (MGUS) to malignant multiple myeloma (MM).

More specifically, monoclonal gammopathies of clinical significance (MGCS) are pathogenic M-proteins linked to a variety of clinical presentations. Unlike MGUS, the proliferation of the clonal plasma cells in MGCS is responsible for organ damage and its related myriads of clinical symptoms and signs. Of note, monoclonal gammopathies of renal significance (MGRS) are the best known among the category of MGCS [1].

In the same context, MGCS-associated myopathies are a group of rare disorders in which the clonal plasma cells are responsible for muscle damage. The latter group includes amyloid light chain (AL) amyloidosis-associated myopathy, sporadic late-onset nemaline myopathy with monoclonal gammopathy (SLONM-MG), and the exceptional non-amyloid light chain deposition disease (LCDD)-associated myopathy [1-6].

More recently, a novel acquired disease within MGCS-associated myopathies was recognized and is defined by the presence of an accumulation of glycogen in the muscle in association with MGCS. This acquired glycogen storage myopathy (GSM) was reported to respond to immunotherapy and/or chemotherapy [7-10].

The goal of this narrative review is to describe the MGCS-associated myopathies by highlighting the pathogenesis, clinical manifestations, relevant investigational results, therapeutic management, and prognosis of these acquired myopathies. The common pitfalls potentially leading to the overlook of MGCS-associated myopathies are also emphasized in this article. We aim, through this review, to raise awareness among physicians, especially neurologists and internists, about the MGCS-associated myopathies due to their difficult diagnosis, high disability, and treatability. Table 1 summarizes the clinical presentations, relevant investigations, therapeutic strategies, and prognosis of MGCS-associated myopathies.

**Table 1:** Clinical manifestations, relevant investigations, therapeutic management, and prognosis of MGCS-associated myopathies. MGCS: monoclonal gammopathy of clinical significance; GSM: glycogen storage myopathy; M-protein: monoclonal protein; CK: creatine kinase; MRI: magnetic resonance imaging; EMG: electromyography; FP: fibrillation potentials; ASCT: autologous stem cell transplant; AL: amyloid light chain; LCDD: light chain deposition disease; SLONM: sporadic late-onset nemaline myopathy; MM: multiple myeloma; M-protein: monoclonal protein; IHC: immunohistochemistry; EM: electron microscopy; IVIG: intravenous immunoglobulin; VAMMGS: vacuolar myopathy with monoclonal gammopathy and stiffness; POEMS syndrome: polyneuropathy, organomegaly, endocrinopathy, monoclonal protein, skin changes; nm: nanometers; FLC: free light chains

Characteristic	AL amyloidosis-associated myopathy	Non-amyloid LCDD-associated myopathy	SLONM-MGCS	MGCS-associated GSM
Clinical manifestations of muscle involvement	Proximal atrophy, weakness, pseudohypertrophy, and myalgia. Dysphagia, macroglossia, jaw claudication, and hoarseness.	Similar to AL amyloidosis-associated myopathy.	Axial and proximal muscle atrophy and weakness, severe weakness of neck extensors (dropped head syndrome), dysphagia, and respiratory insufficiency. In some cases, cardiomyopathy is present.	Subacute proximal myopathy with marked stiffness (VAMMGS phenotype), or progressive axial, proximal, and distal, asymmetric, and painless weakness (without stiffness).
Systemic manifestations	Heart failure, nephrotic syndrome, hepatomegaly, carpal tunnel syndrome, and periorbital purpura.	Multiple organs may be involved, with the kidney being the most involved organ. MM should be ruled out.	No systemic involvement outside the muscle (skeletal and cardiac muscle).	No systemic manifestations.
Serum CK levels	Moderately elevated, but can be normal in more than half of cases.	Normal or moderately elevated.	Most cases: normal, can be slightly elevated in some patients.	Normal or elevated.
EMG	Myopathic pattern, spontaneous activity at rest (FP).	Myopathic pattern, spontaneous activity at rest.	Non-specific myopathic pattern, abundant spontaneous activity at rest (FP).	Myopathic pattern, frequent spontaneous activity at rest.
MRI of muscles	Edema and atrophy of muscles, reticulation in the subcutaneous fat, can be more suggestive.	It can be similar to AL amyloidosis-associated myopathy.	Preferential involvement of neck extensors, paraspinal, gluteal, hamstring, and soleus muscles.	Insufficient data
M-protein	Mostly lambda monotypic light chains.	Often, kappa monotypic light chains.	Mostly IgG kappa or IgG lambda. Exceptionally, isolated monoclonal FLC.	Often IgG kappa, sometimes IgA kappa.
Muscle biopsy findings	Amyloid deposits confirmed by Congo red staining. Immunostaining defines the type of amyloid deposits. EM: Non-branching fibrils arranged in beta-pleated sheets.	The deposits of monotypic light chains, non-congophilic (Congo red negative), on muscle biopsy. IHC is critical to confirm the deposits of monoclonal light chains. EM: no fibrils, granular, electron-dense material.	Nemaline rods revealed as sarcoplasmic aggregates with reddish purple color on the modified Gomori trichrome staining. Rods are positively stained on IHC for Z-band proteins, such as alpha-actinin and myotilin. EM: confirmation of rods as high electron density, thread-like structures.	Accumulation of glycogen, free and/or in vacuoles, within muscle fibers. Myofibrillar disintegration and abnormal mitochondria can be observed on EM.
Therapeutic management	ASCT, if not suited for ASCT: bortezomib, daratumumab.	ASCT and bortezomib-based regimens.	IVIG can be proposed as a first-line treatment; however, ASCT is the most effective therapy (according to many experts).	Non-chemotherapeutic therapies (IVIG, steroids, and immunosuppressive drugs) or chemotherapy-based therapies.
Prognosis	Muscle weakness improves in parallel with the removal of M-protein; the overall prognosis is determined by the number of involved systems (especially the heart).	Muscle weakness improves in parallel with the eradication of M-protein; the overall prognosis is determined by the extent of organ involvement (renal and cardiac) and the hematologic response.	M-protein is associated with a worse prognosis in SLONM, especially rapid progression. Fatal respiratory failure occurs within five years of onset if no treatment is provided. The best neurological improvement is tied to deep hematological remission.	The eradication of M-protein is associated with significant neurological improvement.

## Review

Methodology

This narrative review was developed to describe the definition, pathophysiology, clinical manifestations, relevant investigations, therapeutic strategies, and prognosis of MGCS-associated myopathies. Search terms on PubMed and Google Scholar included various combinations of "monoclonal gammopathy of clinical significance", "myopathy", "amyloidosis AL-associated myopathy", "light chain deposition disease-associated myopathy", "sporadic late-onset nemaline myopathy", and " monoclonal gammopathy-associated glycogen storage myopathy". A strict timeframe was not established for this narrative review, though we privileged the relevant literature for the past 10 years. We included case reports, case series, and reviews related to MGCS-associated myopathies.

Definition of MGCS-associated myopathies

MGCS are characterized by two features: the existence of a clone in the bone marrow, which often produces an M-protein, and the clinical manifestations linked to the M-protein. Notably, MGCS mainly affects the kidney, nerve, and skin [11]. MGCS-associated myopathies are a rare group of diseases in which the M-protein causes significant muscle damage.

In this context, AL amyloidosis-associated myopathy and SLONM-MG are well recognized within the category of MGCS-associated myopathies [1]. Other rarest MGCS-associated myopathies are still overlooked, such as non-amyloid LCDD-associated myopathy and the recently described MGCS-associated GSM [2,10].

Physiopathology of MGCS-associated myopathies

The deposition in organs of whole or part of the M-protein represents the primary pathogenic mechanism in MGCS. Autoantibody activity against a tissue antigen, synthesis of immune complexes, and complement activation are additional mechanisms involved in MGCS. Significant cytokine secretion can also be induced by the clone. Nevertheless, in some situations, the pathogenesis is still unknown [12].

The specific pathophysiology of MGCS-associated myopathies is not fully understood, and the pathogenic link between muscle damage and M-protein remains unclear. Nonetheless, the dysregulation of the immune system, induced by the clonal proliferation of plasma cells, is considered to play a key role in the pathogenesis of MG-associated myopathies. This is underpinned by the improvement of muscle weakness occurring alongside the eradication of serum M-protein by chemotherapy [13].

Some authors speculated that the deposition of light and heavy chains of M-proteins has myotoxic effects on skeletal muscle. This mechanism may explain the pathogenesis of AL amyloidosis-associated myopathy and non-amyloid LCDD-associated myopathy. However, in SLONM-MG, the deposition of light and heavy chains was rarely reported. Therefore, this mechanism does not explain the pathogenesis of such cases in which the deposition of M-proteins is lacking. In SLONM-MG, although the precise role of monoclonal gammopathy is unclear, it is assumed that M-protein may exert a direct toxic effect on muscle fibers or induce immune-mediated muscle damage. In MGCS-associated GSM, we hypothesize that the interaction between the M-protein and the muscle could, via a toxic effect of the M-protein, disrupt the pathways of glycogen metabolism and therefore lead to an accumulation of glycogen within the muscle fibers [10,14].

AL amyloidosis-associated myopathy

AL amyloidosis-associated myopathy is well identified within the category of MGCS-associated myopathies. The deposits of misfolded light chains of M-protein in various organs, as amyloid fibrils, lead to systemic AL amyloidosis [1]. The heart, kidneys, liver, and peripheral nervous system are the most affected organs in AL amyloidosis.

The incidence of skeletal muscle involvement in systemic AL amyloidosis is very low and estimated at 1.5%. Clinically, AL amyloidosis-associated myopathy commonly manifests as weakness (often proximal predominant, but sometimes spreading to distal parts of limbs), muscle atrophy, myalgia, pseudohypertrophy, dysphagia, macroglossia, jaw claudication, and hoarseness. In exceptional cases, the myopathy may be the revealing manifestation of systemic AL amyloidosis [15].

In paraclinical investigations, the serum free light chains (FLCs) test is the most sensitive method for assessing the monoclonal FLC, and bone marrow biopsy should be performed to rule out the possibility of an associated MM. Creatine kinase (CK) levels can be moderately elevated; still, levels may be normal in more than half of the patients. Electromyography (EMG) can reveal a non-specific myopathic pattern or sometimes associated spontaneous activity (fibrillation potentials and positive sharp waves). Echocardiographic abnormalities such as altered myocardial relaxation, thickening of ventricular walls, pericardial effusion, and a restrictive filling pattern can be observed in approximately two-thirds of the patients. Although edema and atrophy in muscle imaging were reported, some more suggestive patterns on magnetic resonance imaging (MRI) in amyloid myopathy were described, such as reticulation in the subcutaneous fat and non-significant signal abnormalities in muscles.

**Figure 1:**
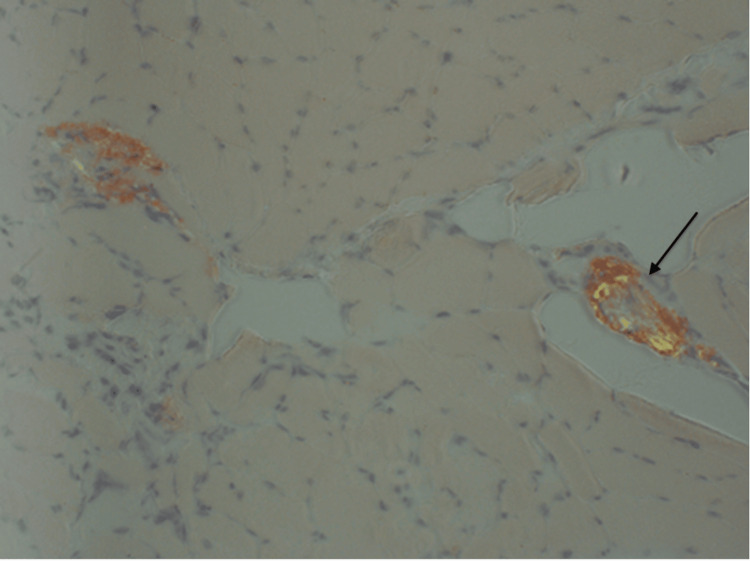
Muscle biopsy under polarized light revealing vascular amyloid deposits with characteristic apple-green birefringence in a patient with AL amyloidosis-associated myopathy (arrow). AL: amyloid light chain Source: Reference [16].

**Figure 2:**
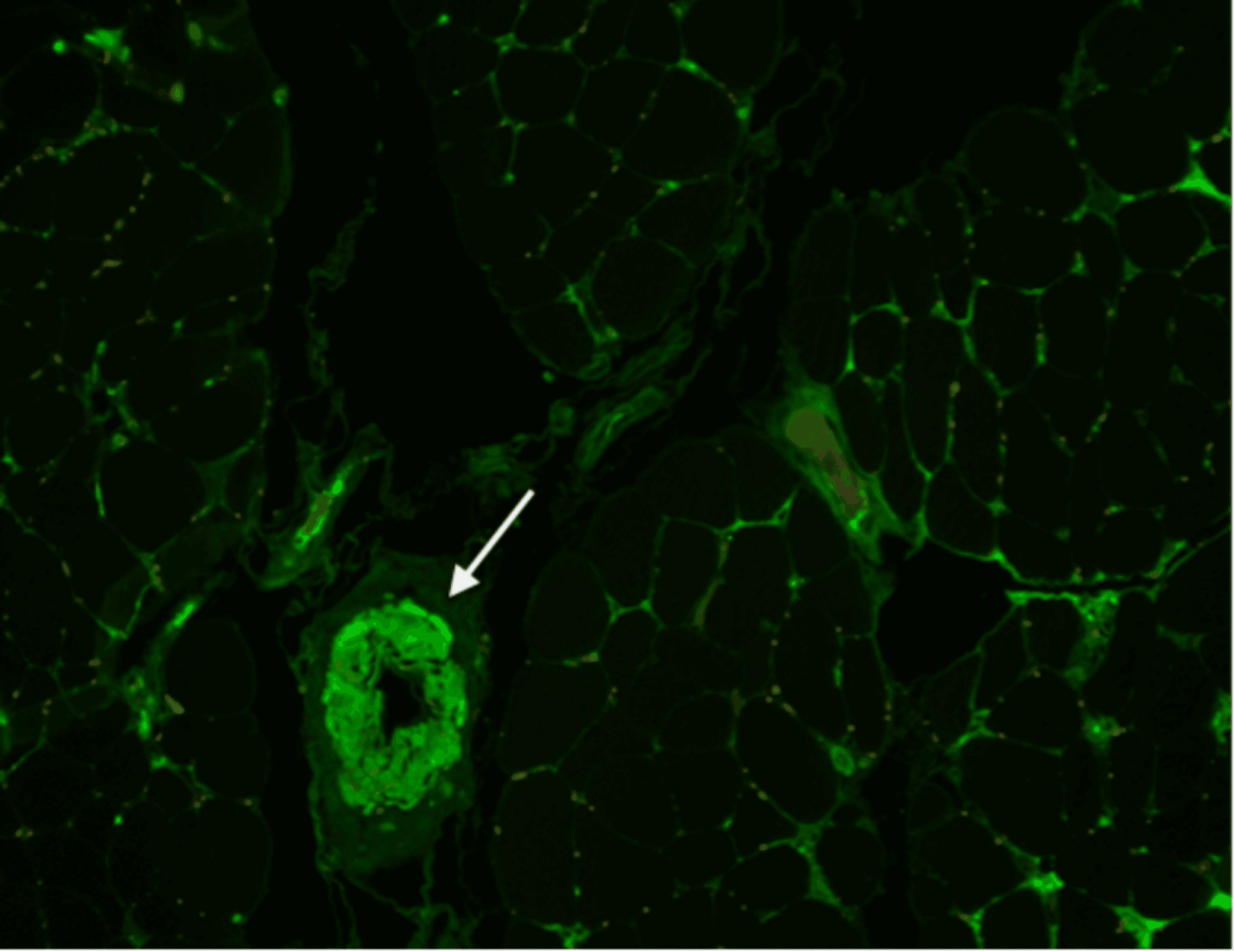
Muscle biopsy showing immunofluorescence microscopy with positive lambda light chain staining in a patient with AL amyloidosis-associated myopathy (arrow). AL: amyloid light chain Source: Reference [16].

However, the key examination to confirm AL amyloidosis-associated myopathy is a muscle biopsy. The hematoxylin and eosin (H&E) staining can be unremarkable or may reveal non-specific lesions such as atrophic, regenerating, and necrotic myofibers. The amyloid deposits in the intramuscular blood vessel walls, perimysium, and endomysium are confirmed by Congo red staining (the characteristic apple-green birefringence under polarized light) and/or electron microscopic study (non-branching fibrils arranged in β pleated sheets). The type of amyloid should be determined using immunohistochemistry (IHC) or mass spectrometry (Figures 1, 2).

The communication between the clinician and the pathologist is crucial. Indeed, the failure to routinely incorporate Congo red staining on muscle biopsy examination may lead to a significant number of false-negative results and thus missing the diagnosis of amyloid myopathy [1,15-17].

Autologous stem cell transplant (ASCT) is the preferred treatment for systemic AL amyloidosis. This therapeutic procedure aims to achieve complete hematologic remission by eradicating the M-protein. Other therapeutic alternatives, such as bortezomib and daratumumab, are best suited for patients who are not candidates for ASCT. Median overall survival of systemic AL amyloidosis is 32 months. Factors associated with lower survival were involvement of more than two organs, cardiac involvement, and absence of ASCT [1,15,16].

Non-amyloid LCDD-associated myopathy

LCDD is characterized by the deposition, in various tissues, of non-amyloid monoclonal immunoglobulin. These deposits are of granular form and non-congophilic (negative Congo red staining). The kidneys are the most affected organ in LCDD; however, any organ can be involved in this systemic disease, such as the liver, the heart, and the nervous system. MM is more frequently associated with LCDD than with AL amyloidosis and should therefore be ruled out in the context of LCDD [18].

**Figure 3:**
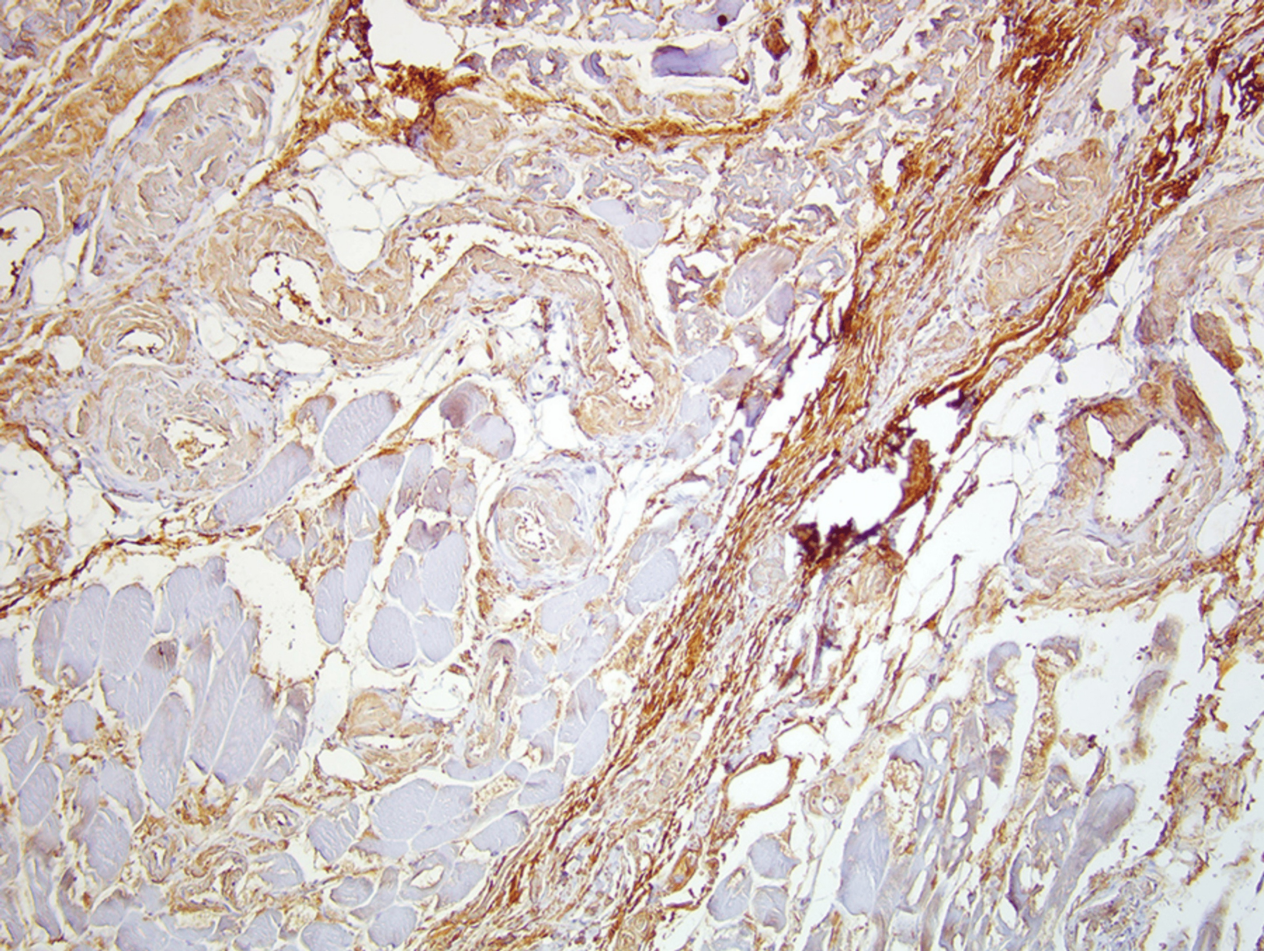
Muscle biopsy showing IHC revealing intense deposits of monotypic lambda light chains (brown) in a patient with non-amyloid light chain deposition disease-associated myopathy. IHC: immunohistochemistry Source: Reference [2].

The involvement of skeletal muscle, in the setting of non-amyloid LCDD, was exceptionally reported in the literature and may even present as the initial manifestation of the disease [2,3,4,19]. In one of these reports, the authors described the co-existence of amyloid angiopathy and non-congophilic kappa light chain deposition in the same muscle [3]. The endomysium along sarcolemmas, vessel walls, and perimysium are the main sites of deposition in LCDD. Indeed, the basement membranes are preferentially involved in monoclonal immunoglobulin deposition disease (MIDD), probably due to a certain affinity of M-proteins for some basement membrane components (Figure 3) [2,20].

Similar to systemic AL-amyloidosis, LCDD can affect multiple organs and systems, though renal involvement is the most common in LCDD. Nevertheless, the critical difference between the two MIDD lies in its histopathological ground. In non-amyloid LCDD-associated myopathy, the deposits of monoclonal light chains are by nature non-congophilic (Congo red negative) on muscle biopsy. Therefore, in such cases, the use of IHC on muscle biopsy sections is critical to unveil the deposition of non-congophilic monoclonal light chains characteristic of LCDD. Moreover, if electron microscopy is available, it will reveal the granular deposits in LCDD, which are different from the non-branching, fibrillar deposits in AL amyloidosis.

We assume that non-amyloid LCDD-associated myopathy is very overlooked among clinicians. Even if Congo red staining is included in a muscle biopsy study, a negative result could lead to the dismissal of MIDD because systemic AL amyloidosis is the archetype of such diseases. Thus, clinicians need to be aware of non-amyloid LCDD-associated myopathy, especially when amyloid myopathy is suspected, and muscle biopsy is Congo red-negative. The use of IHC on such cases may be critical in confirming the diagnosis of non-amyloid LCDD-associated myopathy [2,19].

ASCT and bortezomib-based regimens appear to have reasonable safety and efficacy for LCDD. The prognosis of LCDD is variable, with a median survival of approximately four years, though some studies report longer median survival [21,22].

Sporadic late-onset nemaline myopathy with monoclonal gammopathies of clinical significance (SLONM-MGCS)

SLONM is a rare, acquired, late-onset muscle disorder with subacute progression, characterized by proximal muscle weakness and atrophy, and the presence of nemaline rods (thread-like structures) in myofibers. Acquired SLONM should be separated from the congenital nemaline myopathies, associated with several genetic mutations, and usually beginning in childhood. The myopathy, in SLONM, usually appears after the fourth decade with a slight male predominance. Of note, MGCS and HIV infection are the most common associations with SLONM. When associated with an MGCS, patients with SLONM may have an unfavorable outcome if untreated [5]. The pathogenesis of SLONM-MGCS is not understood, but histopathological studies have demonstrated that nemaline rods arise from the degradation of Z-bands of the sarcomeres with insufficient autophagy. Some authors assume that nemaline rods represent secondary pathological changes induced by the toxic effect of the M-protein on the sarcomeres of the myofibers [23].

**Figure 4:**
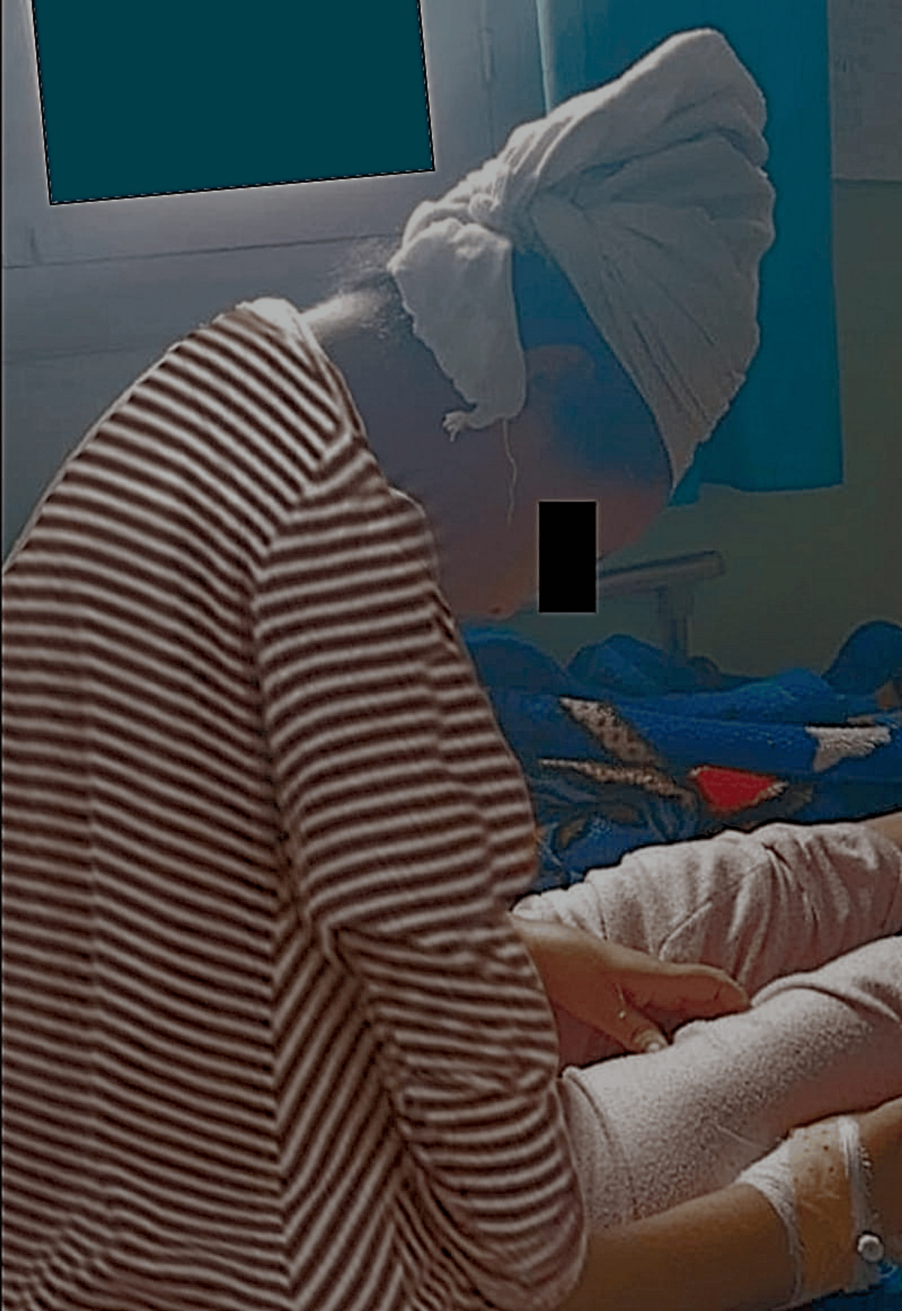
Dropped head syndrome in a patient with SLONM-MGCS (from a personal, unpublished case). SLONM-MGCS: sporadic late-onset nemaline myopathy associated with monoclonal gammopathies of clinical significance

Notably, in contrast to AL amyloidosis-associated myopathy and LCDD-associated myopathy, there is no systemic involvement outside the muscle (skeletal and cardiac muscle) in this disease. In SLONM-MGCS, the weakness and atrophy involve axial and limb-girdle muscles. Distal muscles may also be involved. Bulbar manifestations such as dysphagia and respiratory insufficiency can occur during the evolution of the disease and indicate a negative prognosis. Severe neck extensor muscle weakness or dropped head syndrome is a characteristic feature in SLONM-MG patients and may be observed in more the half of the patients (Figure 4).

Respiratory insufficiency is the main cause of mortality in these patients [24]. Cardiac muscle involvement, often with mild severity, was rarely reported in SLONM-MGCS [25]. However, cardiomyopathy can be an early and life-threatening complication of SLONM-MG, requiring effective management with heart failure drugs. Interestingly, cardiomyopathy, similarly to skeletal muscles, responded to chemotherapy in SLONM-MG [26].

Serum CK levels are usually normal or sometimes slightly elevated. EMG mostly shows a non-specific myopathic pattern with frequent abnormal spontaneous activity (fibrillation potentials). Sometimes, mixed changes (neuropathic and myopathic) or even a pure neuropathic pattern can be revealed in EMG studies [5]. MRI may reveal preferential involvement of certain muscles, particularly those located in posterior compartments, such as neck extensors, gluteal, hamstring, and soleus [27]. The combination of different assays is recommended for screening of MG in patients with SLONM.

**Figure 5:**
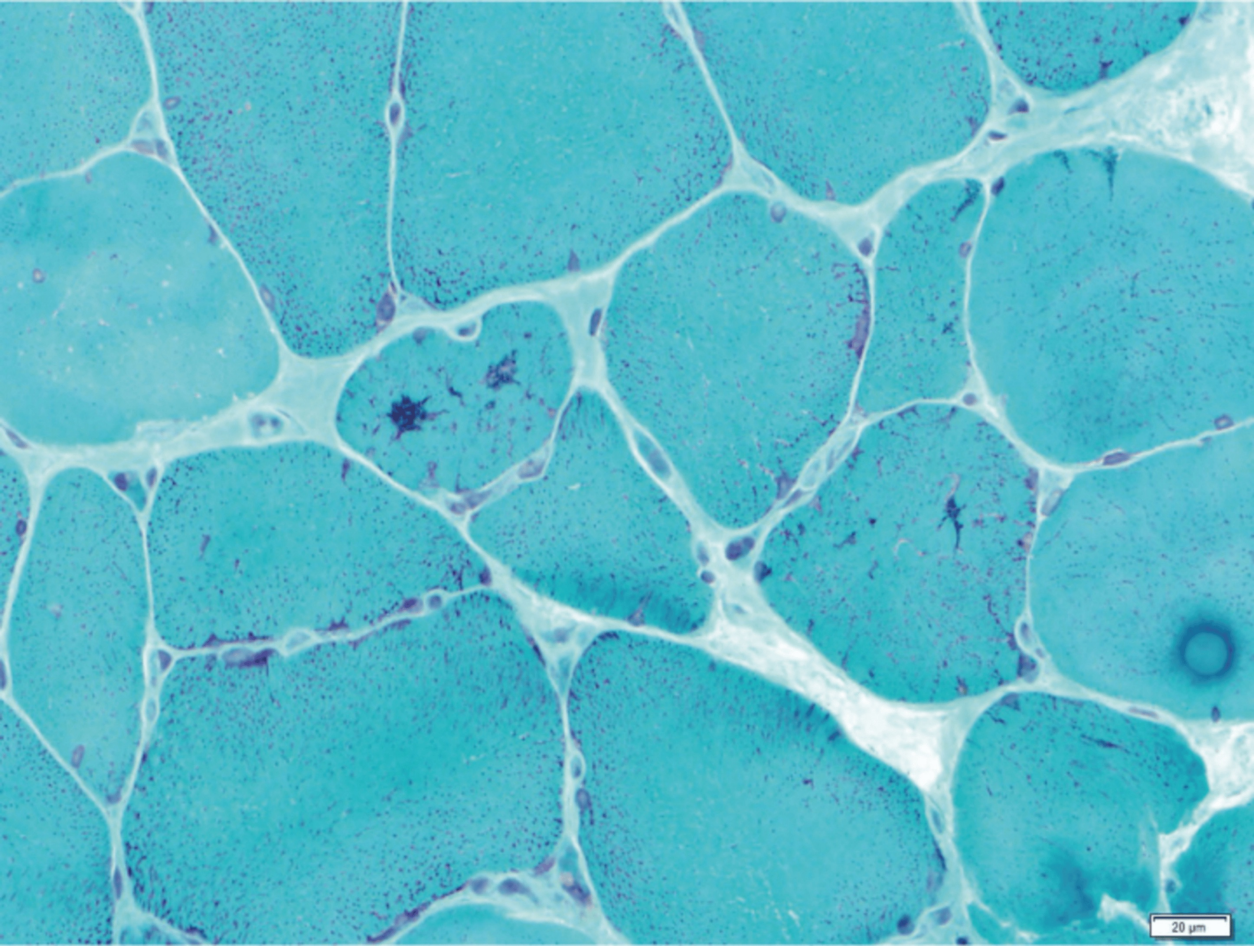
Muscle biopsy with modified Gomori trichrome staining revealing rods as sarcoplasmic aggregates with a reddish-purple color. Source: Reference [28].

The muscle pathology is crucial for the confirmation of the diagnosis of SLONM. The characteristic findings are nemaline rods in myofibers. These rods are usually smaller than 1μm and occupy the whole sections of atrophic fibers. The proportion of myofibers bearing nemaline rods is variable among patients and among different muscle samples of the same patient. Observation of trichromatically stained frozen sections with higher magnification is recommended. Nemaline rods are revealed as sarcoplasmic aggregates with reddish purple color on the modified Gomori trichrome staining (Figure 5) [28].

**Figure 6:**
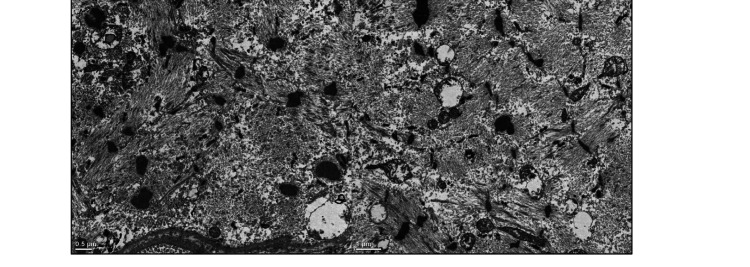
Muscle biopsy with electron microscopy showing numerous nemaline rods as high-electron-density, thread-like structures in a patient with SLONM-MGCS. SLONM-MGCS: sporadic late-onset nemaline myopathy associated with monoclonal gammopathies of clinical significance Source: Adapted from reference [25] with permission from Elsevier Masson.

Nemaline rods are positively stained on IHC for Z-band proteins, such as alpha-actinin and myotilin, because nemaline rods arise from disintegrated Z bands. In the ultrastructural study, rods are morphologically demonstrated by the high electron density similar to Z bands and the presence of thread-like structures (Figure 6) [5,24,25].

Other non-specific pathologic features in SLONM-MG are atrophic fibers, moderate inflammatory infiltrates, myofibrillar disintegration, mitochondrial abnormalities, core-like areas, and lobulated fibers [24,25]. Due to the patchy distribution of nemaline rods within muscles, MRI can be very helpful in selecting the affected muscles for targeted muscle biopsy and thus avoiding false-negative results.

It was suggested that the presence of M-protein heralds a worse prognosis in SLONM, with particularly rapid progression in those patients. Without treatment, fatal respiratory insufficiency, within five years from the onset, was reported among patients with SLONM-MG [5,29]. Two approaches are used to treat SLONM-MGCS: (1) immunosuppressive therapy (steroids, steroid-sparing agents, intravenous immunoglobulins (IVIG), and plasmapheresis/plasma exchange) or (2) chemotherapy followed by ASCT. However, based on the experience of many authors, it is assumed that chemotherapy followed by consolidative ASCT is more effective than immunotherapy and should be considered as a preferred approach for patients with SLONM-MGCS. This is supported by the best clinical response and overall survival achieved in patients with SLONM-MGCS receiving chemotherapy-based treatment [13,24,29,30]. Indeed, in a large cohort of 53 patients with SLONM-MG, the authors of this study compared the effectiveness of treatment and outcomes in patients receiving chemotherapy versus those receiving non-chemotherapeutic therapies. Neurological improvement in the non-chemotherapy group (N = 25) was observed in 52% of patients, whereas it was seen in 86% of patients in the chemotherapy group (N = 28). Moreover, the mean time to best response in the chemotherapy group was significantly shorter (eight months) than in the non-chemotherapy group (21 months), and the overall survival was higher in patients in the chemotherapy group [30].

MGCS-associated GSM

More recently, a novel acquired disease within MGCS-associated myopathy was recognized and is defined by the presence of an accumulation of glycogen in the muscle in association with MGCS. This acquired GSM was reported to respond to immunotherapy and/or chemotherapy [7-10].

In 2019, the first three cases of MGCS-associated GSM were described by Allenbach et al. [7]. Then, the same cases were prospectively studied and reported in a recent report [9]. These three patients developed subacute myopathy in addition to marked stiffness. The most relevant pathologic finding was the presence of vacuoles filled with glycogen in the muscle biopsies. These cases all had MG and responded to IVIG and immunosuppressive therapies. The authors termed this MG-associated GSM as vacuolar myopathy with monoclonal gammopathy and stiffness (VAMMGS) [7,9].

In 2023, in the same setting, Soontrapa et al. reported the case of a female patient with polyneuropathy, organomegaly, endocrinopathy, M-protein, and skin changes (POEMS) syndrome. The patient also developed mild proximal myopathy. The muscle biopsy demonstrated fibers with glycogen-filled vacuoles, with additional rare myofibers containing polyglucosan bodies. The patient had a biclonal gammopathy, and myopathy improved after the ASCT [8].

**Figure 7:**
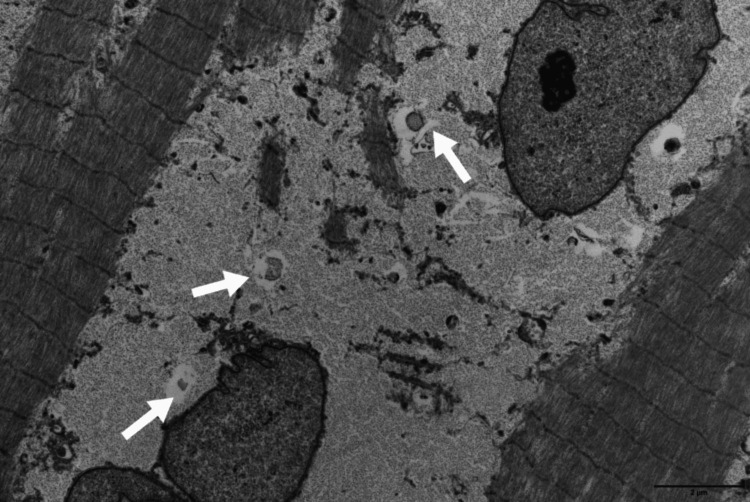
Muscle biopsy with electron microscopy revealing accumulation of glycogen between myofibrils (arrows) in a patient with MGCS-associated GSM. MGCS-associated GSM: monoclonal gammopathy of clinical significance-associated glycogen storage myopathy Source: Reference [10].

In our recently published case, the patient developed a rapidly progressive, painless weakness of all limbs. The topographic pattern of the weakness was asymmetric and involved both the proximal and distal muscles of the limbs. The axial muscles were also severely affected. Unlike the VAMMGS, our patient did not develop stiffness, and the serum CK was within normal range. The EMG revealed a myopathic pattern in the affected muscles with abnormal spontaneous activity (fibrillation potentials and positive sharp waves). IgA kappa MG and free monoclonal kappa chains were revealed in the serum. Muscle biopsy showed a severe GSM (Figure 7).

There was no evidence for hereditary glycogen metabolic disorder throughout genetic studies. The protocol of eight cycles of melphalan-dexamethasone has led to a significant hematological response in parallel to the improvement of muscle weakness in our patient [10].

Relying on our case and the previous reports, we assume that the features (clinical pattern of weakness, CK levels, and EMG) of MGCS-associated GSM are variable. More cases are needed in the future to better elucidate this novel acquired muscle disease. Although aggressive chemotherapy was preferred in our case, we assume that the most appropriate therapeutic strategy (chemotherapy versus immunotherapy) remains to be clarified in MGCS-associated GSM. More cases are therefore needed to draw clear therapeutic approaches in this rare disease.

Common pitfalls in the diagnosis of MGCS-associated myopathies

Since the burden of the MGCS associated with neuromuscular diseases is low, relying solely on serum protein electrophoresis (SPE) can lead to a significant percentage of false-negative results and, hence, missing the presence of the M-protein. For a more sensitive screening for the M-protein, clinicians should demand a comprehensive set of tests. In addition to SPE, immunofixation of serum and urine, serum FLC kappa and lambda, and even blood mass spectrometry (if available) should be included when clinicians suspect a myopathy due to MGCS [1]. To exclude an underlying MM, bone marrow aspiration and biopsy with IHC or flow cytometry should be added.

In MGCS-associated myopathies, repeated muscle biopsies and/or additional techniques on muscle biopsy are sometimes required to establish the diagnosis. In AL-associated myopathy, amyloid angiopathy can be overlooked due to biopsy sampling error. Indeed, cases of amyloid myopathy have been reported to necessitate as many as four muscle biopsies to confirm the diagnosis. In SLONM-MGCS, the confirmation of the diagnosis may also require repeated muscle biopsies. Indeed, the uneven distribution of nemaline rods within different muscles of the same patient can lead to false-negative results in histopathological studies. Moreover, it was suggested that the appearance of nemaline rods may be delayed several months after the onset of the clinical weakness [3,13,29].

In some difficult cases, in which the nemaline rods are very small, optic microscopic study using the modified Gomori trichrome staining may fail to detect these nemaline bodies. In such cases, when suspicion of SLONM is high, electron microscopic examination may reveal very small rods. In non-amyloid LCDD-associated myopathy, negative Congo red staining of the muscle biopsy would overlook the diagnosis. Therefore, clinicians should demand the IHC on muscle biopsy if they suspect amyloid myopathy with negative Congo red staining on muscle biopsy [2,13,19,25].

## Conclusions

MGCS-associated myopathies encompass an array of diseases in which a highly toxic M-protein plays a key role in muscle damage. The accurate diagnosis of MGCS-associated myopathies relies on comprehensive and sensitive methods to detect the M-protein. The confirmation of these myopathies relies on muscle biopsy, sometimes repeated, and on using various techniques to search for distinctive histopathological features. In the future, protocols of chemotherapy (with or without ASCT) should be clearly defined for MGCS-associated myopathies.
